# SodiUm SeleniTe Adminstration IN Cardiac Surgery (SUSTAIN CSX-trial): study design of an international multicenter randomized double-blinded controlled trial of high dose sodium-selenite administration in high-risk cardiac surgical patients

**DOI:** 10.1186/1745-6215-15-339

**Published:** 2014-08-28

**Authors:** Christian Stoppe, Bernard McDonald, Steffen Rex, William Manzanares, Richard Whitlock, Stephen Fremes, Robert Fowler, Yoan Lamarche, Patrick Meybohm, Christoph Haberthür, Rolf Rossaint, Andreas Goetzenich, Gunnar Elke, Andrew Day, Daren K Heyland

**Affiliations:** Department of Anesthesiology, University Hospital of the RWTH Aachen, Pauwelsstraße 30, 52074 Aachen, Germany; Division of Cardiac Anesthesiology and Critical Care Medicine, University of Ottawa Heart Institute Ottawa, 40 Ruskin Street, Ottawa, Ontario K1Y 4W7 Canada; Department of Anesthesiology & Department of Cardiovascular Sciences, University Hospitals Leuven, Herestraat 49, 3000 Leuven, Belgium; Department of Critical Care Medicine, Universidad de la República, UDELAR, Avda Italia s/n esq. Las Heras, CP, 11600, Montevideo, Uruguay; Department of Surgery, Population Health Research Institute, McMaster University, 1280 Main St W, Hamilton, ON L8S 4L8 Canada; Division of Cardiac Surgery, Schulich Heart Centre, Sunnybrook Health Sciences Centre, 2075 Bayview Ave, Toronto, Ontario M4N 3M5 Canada; Department of Medicine and Department of Critical Care Medicine, Sunnybrook Health Sciences Centre, 2075 Bayview Ave, Toronto, Ontario M4N 3M5 Canada; Department of Surgery and Critical Care, Montreal Heart Institute and Hôpital du Sacré-Cœur de Montréal, Institut de cardiologie de Montréal, 5000 Bélanger E Montréal, Quebec, H1T1C8 Canada; Department of Anaesthesiology, Intensive Care Medicine and Pain Therapy, University Hospital Frankfurt, Theodor-Stern-Kai 7, 60590 Frankfurt am Main, Germany; Department of Anaesthesiology and Intensive Care Medicine, Hirslanden Clinic, Witellikerstrasse 40, 8032 Zurich, Switzerland; Department of Thoracic, Cardiac and Vascular Surgery, University Hospital, RWTH Aachen, Pauwelsstraße 30, 52074 Aachen, Germany; Department of Anaesthesiology and Intensive Care Medicine, University Medical Center Schleswig-Holstein, Campus Kiel, Arnold-Heller-Str. 3, 24105 Kiel, Germany; Clinical Evaluation Research Unit, Kingston General Hospital, 76 Stuart Street, Kingston, Ontario K7L2V7 Canada

**Keywords:** Selenium, Inflammatory response, Oxidative stress, Antioxidant capacity, Myocardial ischemia/reperfusion, Postoperative organ failure

## Abstract

**Background:**

Cardiac surgery has been shown to result in a significant decrease of the antioxidant selenium, which is associated with the development of multiorgan dysfunction and increased mortality. Thus, a large-scale study is needed to investigate the effect of perioperative selenium supplementation on the occurrence of postoperative organ dysfunction.

**Methods/Design:**

We plan a prospective, randomized double-blind, multicenter controlled trial, which will be conducted in North and South America and in Europe. In this trial we will include 1,400 high-risk patients, who are most likely to benefit from selenium supplementation. This includes patients scheduled for non-emergent combined and/or complex procedures, or with a predicted operative mortality of ≥5% according to the EuroSCORE II. Eligible patients will be randomly assigned to either the treatment group (bolus infusion of 2,000 μg sodium selenite immediately prior to surgery, followed by an additional dosage of 2,000 μg at ICU admission, and a further daily supplementation of 1,000 μg up to 10 days or ICU discharge) or to the control group (placebo administration at the same time points).

The primary endpoint of this study is a composite of 'persistent organ dysfunction’ (POD) and/or death within 30 days from surgery (POD + death). POD is defined as any need for life-sustaining therapies (mechanical ventilation, vasopressor therapy, mechanical circulatory support, continuous renal replacement therapy, or new intermittent hemodialysis) at any time within 30 days from surgery.

**Discussion:**

The SUSTAIN-CSX™ study is a multicenter trial to investigate the effect of a perioperative high dosage sodium selenite supplementation in high-risk cardiac surgical patients.

**Trial registration:**

This trial was registered at Clinicaltrials.gov (identifier: NCT02002247) on 28 November 2013.

**Electronic supplementary material:**

The online version of this article (doi:10.1186/1745-6215-15-339) contains supplementary material, which is available to authorized users.

## Background

Cardiac surgery is performed annually in approximately one million patients worldwide. If current healthcare use and service delivery patterns continue the demand for cardiac surgery is expected to increase on the basis of population growth and ageing [1].

**Figure 1:**
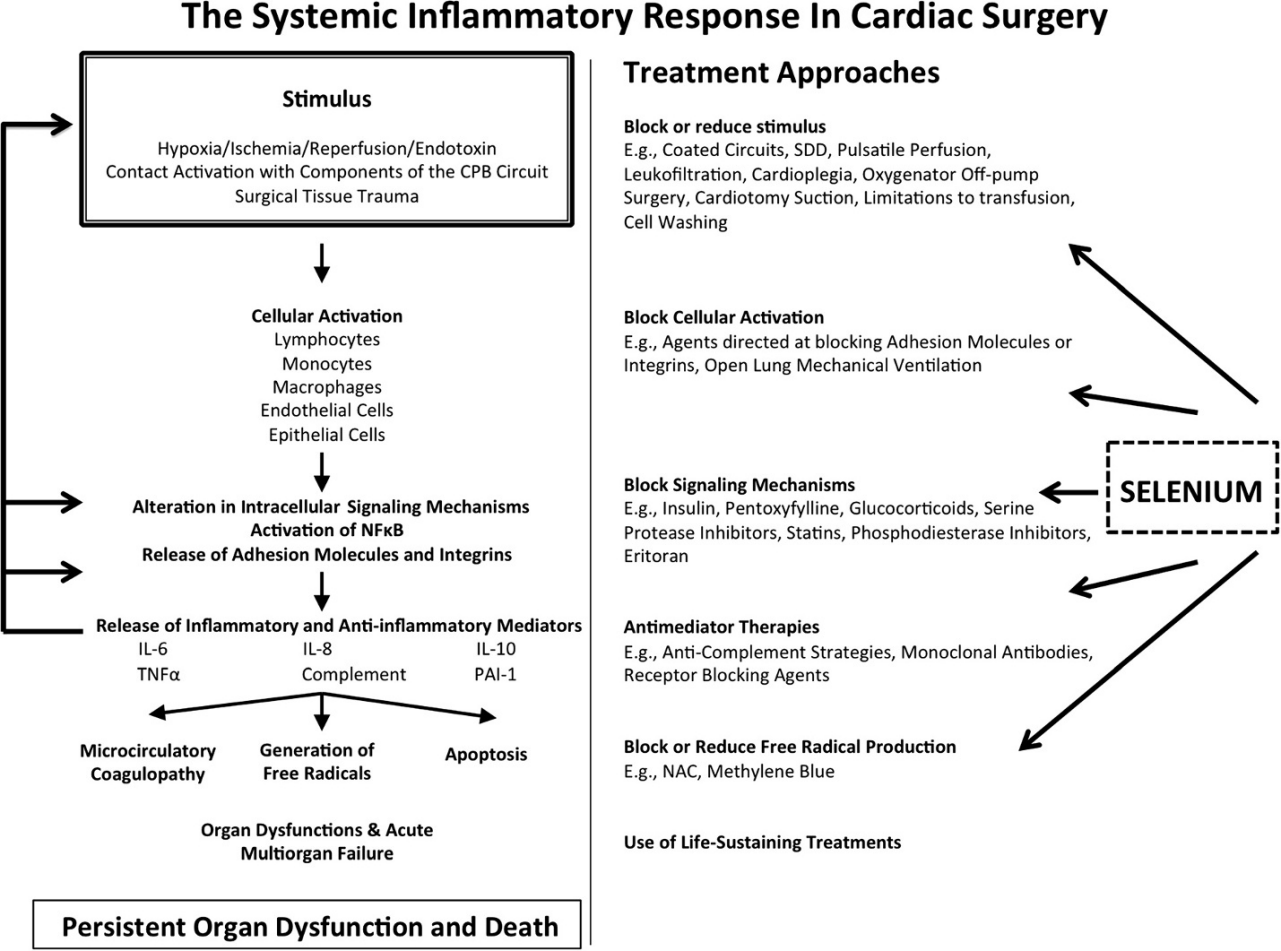
**Multiple stimulus and treatment approaches in cardiac surgery.** The present figure was modified and reproduced with permission from Hall [2]. CPB = cardiopulmonary bypass; IL = interleukin; TNF = tumor necrosis factor; PAI = Plasminogen activator inhibitor; NAC = N-acetylcysteine.

Patients undergoing cardiac surgery with cardiopulmonary bypass (CPB) are exposed to various ischaemic stimuli, resulting from global ischaemic cardioplegic arrest of the heart and/or from embolic events. Cessation of CPB and reperfusion of ischemic vascular beds evokes oxidative stress and triggers an intense inflammatory response, which is associated with endothelial dysfunction, microvascular thrombosis, immune dysfunction, and eventually the potential for injury of virtually all vital organs, including heart, lungs, brain, kidneys and intestines (Figure 1) [2, 3]. In mammals, a sophisticated endogenous defense system protects tissues from oxidative stress. Several enzymes such as catalase, superoxide dismutase and glutathione peroxidase (GPx) are specifically designed to neutralize reactive oxygen species [4]. For these antioxidant (AOX) enzymes, interest in the essential trace elements selenium has arisen as it is involved in multiple steps of intracellular AOX defense [5, 6] and thus can neutralize both reactive oxygen and nitrogen species [4]. Selenium is considered as the cornerstone of the antioxidant defense mechanism and may be one of the most important antioxidants [7]. When incorporated into the various selenoenzymes, selenium increases its antioxidant capacity and influences the inflammatory signaling pathways that modulate reactive oxygen species (ROS) by inhibiting the nuclear factor-kappa b (NF-kB) cascade, resulting in a suppressed production of interleukins and tumor necrosis factor alpha (TNFα) [8].

In addition to critically ill patients, a significant selenium deficiency and hence an insufficient endogenous antioxidant capacity was recently demonstrated among patients after cardiac surgery. Selenium levels fell with increasing CPB time and continued to decrease postoperatively, when compared to selenium levels from a healthy population not undergoing surgery. Importantly, low selenium levels were associated with postoperative complications and the intraoperative selenium decrease showed a predictive accuracy for subsequent development of multi-organ failure [9, 10]. Given those findings and recent results from a non-randomized open-label study which indicated a beneficial effect of perioperative sodium selenite supplementation [11], we aim to study a perioperative sodium selenite supplementation in a large-scale randomized trial, targeting patients who have a high risk of pronounced selenium deficiency. We hypothesize that the therapeutic strategy in this randomized trial may contribute to a lower rate of organ dysfunction postoperatively, and thus, improved health outcomes.

## Methods/Design

### Design

This is a randomized, double-blind (patients and physician), placebo controlled multicenter study, which will be conducted in North America (Canada and United States) and Europe (Belgium, Switzerland and Germany), with an aim to enroll approximately 1,400 patients to evaluate the overall study hypothesis.

### Ethics

The present study (study protocol, patient information and informed consent) is approved by the ethics committee of RWTH Aachen University (Ethik-Kommission an der Medizinischen Fakultät der RWTH Aachen, ethic vote EK 249/13) and Queens University (Kingston, Canada). Furthermore it has been approved by Health Canada and the Bundesinstitut für Arzneimittel und Medizinprodukte (BfArM) in Germany. Written informed consent from the patient and treating anesthesiologist or surgeon will be obtained before enrolment in the study.

### Inclusion criteria

Participants in the trial must be adult patients (>18 years of age) scheduled to undergo elective cardiac surgery with the use of cardiopulmonary bypass (CPB) and cardioplegic arrest that exhibit a high perioperative risk profile as defined by either: 1.) planned valve surgery combined with coronary artery bypass grafting or 2.) multiple valve replacement and/or repair surgeries or 3.) combined cardiac surgical procedures involving the thoracic aorta or 4.) scheduled cardiac surgery with a high perioperative risk profile, defined as a predicted operative mortality of ≥5% (EuroSCORE II).

These high-risk patients have recently been shown to experience an excessive systemic inflammatory response, with the most pronounced decrease of selenium during surgery [10, 12]. Furthermore it has been demonstrated that postoperative selenium blood levels were inversely correlated with duration of CPB. More precisely, the longer the surgical procedure, the more pronounced the postoperative decrease in circulating selenium levels (r = -0.121, *P* <0.05) [9]. In addition, the results of our collaborators and those from a recently published study revealed that the preoperatively assessed EuroSCORE I inversely correlated to the postoperatively measured selenium levels (r = -0.312, *P* <0.01), indicating that this group would be most likely to benefit from perioperative selenium supplementation [9]. These same criteria enabled the identification of patients who were likely to experience a prolonged ICU course [13], which will offer some statistical efficiencies given the higher rate of organ dysfunction and need for prolonged administration of life-sustaining therapies.

Since previous randomized trials in cardiac surgery often lacked generalizability, particularly in the case of elderly patients, we decided to enroll patients in accordance to the recommendations of the American Heart Association Council on Clinical Cardiology without any age restriction [14].

### Exclusion criteria

We will exclude patients who meet any of the following criteria: 1) known hypersensitivity to sodium selenite or to any of the constituents of the solution, 2) severe renal dysfunction as evidenced by preoperative creatinine clearance <50 ml/min and/or preoperative value of serum creatinine level >200 μmol/L, 3) chronic liver disease as evidenced by a preoperative total bilirubin >2 mg/dl or 34 umol/L, 4) disabling neuropsychiatric disorders (severe dementia, severe Alzheimer’s disease, advanced Parkinson’s disease), 5) pregnancy or lactation period, 6.) simultaneous participation in another clinical trial of an experimental therapy (co-enrolment acceptable in observational studies or randomized trials of existing therapies if permitted by both steering committees and local ethics boards), 7) patients undergoing heart transplantation or preoperative planned LVAD insertion or complex congenital heart surgery.

These exclusion criteria will enable us to exclude patients who have the lowest likelihood of deriving benefit from the study intervention, and those at high risk of having an atypical postoperative course (patients undergoing heart transplantation), and thus potentially interfering with the valid determination of our primary endpoint.

### Random allocation of patients

At each participating centre the local coordinating investigator will screen daily all cardiac surgical patients scheduled to undergo cardiac surgery in the near future or on the next day. A screening log will be kept at each site to determine the number of patients meeting the inclusion criteria, those truly eligible patients, those who consent and are randomized, and reasons why potentially eligible patients were not enrolled. Following a full explanation of the nature and purpose of the study, written informed consent will be sought from the patients participating in the study. At the time of enrolment into the study, patients will be randomized to receive either sodium selenite or a matching placebo similar in appearance, consistency, volume, and smell so as to blind patients, investigators and healthcare practitioners as to the nature of the study medication. Patients will be consecutively randomized by a web-based randomization system (concealed and blinded) developed by the Clinical Evaluation Research Unit at the Kingston General Hospital and will be based on the method of permutated blocks of undisclosed random size and stratified by centre.

### Intervention

**Figure 2:**
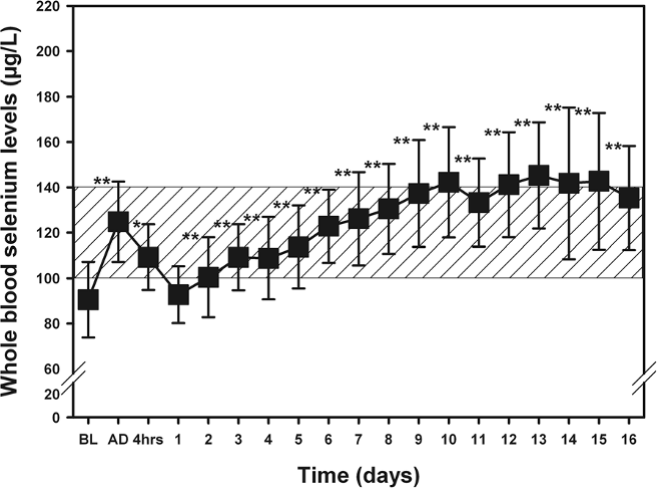
**Perioperative time course of whole blood concentrations of selenium.** The shaded area indicates the reference range for whole blood selenium concentration in Germany. BL: Baseline before induction of anesthesia; AD: admission to the ICU; 4 hrs: 4 hours after admission to ICU. *P <0.05 versus baseline. **P <0.01 versus baseline. This picture was taken from [11]. The shaded area indicates the reference range. Data are given as means ± SD.

Patients will receive daily perioperative treatment of either high-dose sodium selenite or placebo. As with our previously referenced preliminary studies, all patients will receive an intravenous bolus of 2,000 μg sodium selenite (equal to 40 ml prepared solution) or the same volume of normal saline (placebo) within 30 minutes after induction of anesthesia via the central venous catheter. This bolus dose will be completed prior to commencing cardiopulmonary bypass. After termination of surgery, immediately after admission to the ICU, all patients will receive a second bolus of 2,000 μg sodium selenite or placebo, accordingly. This additional postoperative bolus is in response to the postoperative drop in selenium levels observed on day 1 in a recently completed open label trial (Figure 2) [11]. This postoperative drop may be due to sustained oxidative stress, bleeding or transfusions that frequently occur in the first 24 hours of surgery in patients with prolonged CPB. Then, on every subsequent morning (8:00 am) during ICU stay, patients will receive a continuous infusion of 1,000 μg sodium selenite or placebo over 30 minutes via central or peripheral venous access. The daily administration of study solution will continue until death, discharge from ICU (or a step down/intermediate care unit), or for a maximum of 10 days. This dosing regimen was chosen according to efficacy, tolerability and safety that was confirmed in previous supplementation trials in patients with systemic inflammation and cardiac surgery [11, 15, 16]. The intervention drug and placebo solution will be supplied by biosyn Arzneimittel GmBH (Fellbach, Germany), an industry partner that manufactures intravenous sodium selenite in accordance to good manufacture practice (GMP) guidelines and will be provided in such a way as to maintain blinding. Since there exist numerous treatments which may influence the study outcome, we will attempt to guide and standardize at least the management of anaesthesia, intensive and nutritional support (please see Additional file 1).

### Blinding and protection against bias

At the time of enrolment into the study, researchers will be blinded to the treatment assignment (guarding against selection bias). Patients will be randomized to receive either sodium selenite or a matching placebo similar in appearance (consistency, volume and smell) so as to blind patients, investigators and healthcare practitioners as to the nature of the study medication (guarding against performance and detection bias). Recording of relevant clinical data and neurocognitive assessments, will be performed by study personnel blinded to group allocation during the ICU stay (well established CAM-ICU questionnaire, which evaluates the acute onset or fluctuating course, inattention, altered level of consciousness, and disorganized thinking [17]).

Consistent with the intention-to-treat principle, all randomized patients will be included in the analysis. Large numbers of patients lost to follow-up would threaten the validity of this trial. Given that the study occurs in hospital and the majority of the outcomes, including the primary outcome, will be assessed in hospital, we anticipate no loss to follow up in hospital and less than 10% for post-discharge follow-up assessments. With respect to the planned mid-term follow-up portion of the study (secondary endpoint), to guard against attrition bias, we will take the following proactive strategies shown to enhance retention: 1) we will obtain the contact information of a family member in case we cannot contact the patient and/or if the patient is not able to provide information, to obtain the most important patient-centered physical function on SF-36 from the family member [18]; 2) the study coordinator will contact the patient and family member at the time of ICU discharge to build a relationship and reinforce the need for subsequent contact (after three and six months); 3) respondents will be notified of upcoming interviews by means of mail-out reminders; and 4) we will obtain survival status of all patients lost to follow-up from public registries.

The monitoring of sites will be performed by the CERU at the Kingston General Hospital, Ontario, Canada (for the Canadian sites) and by the Clinical Trial Center (CTC) Aachen at RWTH Aachen University, Germany.

### Endpoints

#### Primary outcome

The primary objective of the definitive trial is to investigate the effect of a high-dose sodium selenite perioperative supplementation on clinical outcomes in cardiac surgical patients at high risk for perioperative complications. The selection of a primary clinical outcome for large-scale trials in cardiac surgery is associated with a number of challenges. Options include mortality, length of stay in the ICU and various composite endpoints. Mortality rates are too low in this population to be a responsive primary outcome for this intervention. At the Ottawa Heart Institute, over the past five years in-hospital mortality rate for all patients(elective and emergent) was 3.1%. A total sample size of over 22,000 patients would be required to achieve 80% power to detect a 20% relative risk reduction (RRR) to 2.5% at a two-sided alpha = 0.05. Furthermore, focusing on mortality alone misses the beneficial (or adverse) effect of treatments on morbidity and quality of life. Length of stay in an ICU may be a potential outcome but discharge practices are tremendously variable across units, and are dependent on non-clinical factors such as availability of beds, rendering this outcome less sensitive to detect a treatment effect. A standard composite endpoint of all-cause mortality, myocardial infarction, stroke, renal failure, and prolonged mechanical ventilation could be considered but the inclusion of stroke, particularly due to embolic origins, would not be expected to be modified by our proposed treatment strategy. Moreover, combining myocardial infarction defined by a biochemical change (troponin rise) with an event such as death challenges the validity of the composite endpoint.

A fourth option could be to use persistent organ dysfunction (POD) + death as a composite endpoint. POD is defined as the need for life-sustaining therapies at any time postoperatively (life-sustaining therapy, mechanical ventilation (includes non-invasive ventilation), any vasopressor therapy, mechanical circulatory support, continuous renal replacement therapy or intermittent hemodialysis). The primary endpoint will be the number of days alive and POD-free, which will be evaluated by the composite outcome POD + death within the first 30 days after surgery.

We have validated this endpoint in critically ill patients before and have shown that patients who develop POD are at higher risk for subsequent death and long-term disability or lower quality of life compared to those who do not have POD [19]. In a similar study of cardiac surgery patients, Williams *et al*. showed that the persistence of multiple organ failure identifies a subpopulation of surgical patients that have a high likelihood for death or poor physical function over the subsequent two years [20]. Moreover, given our biological model illustrated in Figure 1, POD would be the most direct outcome and the most sensitive to detecting a treatment effect of sodium selenite (Figure 1).

The 30-day time frame is commonly used in the cardiac surgery literature because there is virtually no loss to follow-up during this period and there are only about 5% of outlying patients who remain alive and dependent on life-sustaining therapy after (sometimes months after) 30 days. Patients who die within 30 days will be given a value of 0 free days. This outcome will have properties similar to 'ventilator free days’ which has been widely accepted in critical care medicine and used in several recent major RCTs [21–23]. Free days will only be counted if they persist for at least 48 hours prior to re-application of life sustaining therapy and are not followed by death within 30 days. We propose to compare the primary outcome between arms using the Wilcoxon rank sum test. The daily proportion of patients alive and free of life-sustaining therapy by arm over the first 30 days will be depicted graphically. The primary analysis will follow the intent-to-treat principle, including all patients in the arm they were randomized to regardless of treatment compliance. The analysis and study reporting will be in accordance with the CONSORT statement [24].

#### Secondary outcomes

Secondary outcomes for the larger scale trial will include perioperative hemodynamic profile (for example mean arterial blood pressure, cardiac output, systemic vascular resistance and so on), cardiovascular complications (such as arrhythmias, cardiac arrest and infarction), ventilator free days, the occurrence of postoperative delirium (assessed by CAM-ICU score) over time, length of stay in the ICU and hospital, re-admission rates, hospital-acquired infections (proven bacteremia) and 30-day mortality. We plan to follow study patients prospectively during the ICU stay, documenting the compliance with the study procedure, reasons for interruptions of study medications and primary and secondary outcomes. Additionally, we will contact patients by telephone at three and six months to assess survival and health-related quality of life (using SF-36 scores) as in our previous antioxidant trial of critically ill patients [25].

Besides these clinical data, we plan to obtain an extensive pharmacokinetic and pharmacodynamic evaluation of our sodium selenite supplementation regimen. Blood samples will be drawn prior to surgery (pre-treatment), after surgery (ICU admission), at the first postoperative day (before start of selenium application) and then daily until ICU discharge or postoperative day 10 to determine whole blood selenium levels (measured by atomic absorption spectroscopy to ensure determination of selenium independent from the compartment). In addition, incorporation of selenium into selenoproteins will be measured through monitoring of GPx activity and selenoprotein P (Sel-P) levels, both of which have been repeatedly shown to correlate with the circulating selenium levels [26, 27]. In addition, we aim to perform genotyping analysis to evaluate the effects of genotype on metabolism, selenium status and health outcomes. The relative ratio between two isoforms of Sel-P has been reported to be influenced by genotype with respect to two single nucleotide polymorphisms (SNPs) in the Sel-P gene, the effect of which was abrogated under conditions of selenium supplementation [27]. Since Sel-P is crucially involved in the systemic selenium transport (blood and tissue), we thus aim to evaluate the significance of the underlying genotype on patients’ response to supplementation and patients’ outcome after surgery. Furthermore, we hope to reveal new insights into the underlying genotype with respect to further selenoproteins.

We shall also aim to assess the extent of oxidative stress and inflammation by measuring MDA-LDL IgM (antibodies against oxidized LDL), asymmetric dimethyl-arginine (ADMA), endogenous peroxidase activity (EPA) and the well-established markers of inflammation: interleukin (IL)-6, IL-10 and TNFα in a substudy. All collected blood samples will be stored at -80°C until final analysis in a central lab at Aachen University. Standard lab tests to detect myocardial ischemia (to detect troponin, for example), inflammation (C-reactive protein) and organ function (creatinine, urea, arterial blood gases, bilirubin and hemoglobin) will be performed according to clinical routine and as clinically indicated.

### Statistical analysis

#### Justification of sample size for the definitive trial

From a database of all patients that underwent cardiac surgery between 1 January 2011 and 31 December 2011 at the University Hospital of Aachen, Germany (n = 1127) and met present inclusion criteria (n = 170), we estimated the control arm distribution of POD free days. In this dataset the mean (SD) POD free days was 23.2 (9.2), factoring in a mortality rate of 4%. The distribution and cumulative distribution of POD free days shows that 4% of the patients died, 6% survived but had 0 POD free days and 58% had 26 to 29 free days. Only 15% of patients had between 1 and 20 free days. These numbers are consistent with the 4% 30-day mortality rate and the 6% not yet free from life-sustaining therapy by 30 days that we observed from the 83 patients seen at the University of Ottawa Heart Institute between July 2012 and June 2013 who met the eligibility criteria of the current calculation. Similar to ventilator free days, the distribution of free days is clearly not Gaussian, with most of its distribution near the minimum and maximum possible values of 0 and 30 respectively.

We performed simulation in order to accurately estimate the actual power of applying the Wilcoxon rank-sum test to scenarios with various effect sizes where the control arm had the same distribution as observed from our German data. The simulation generated data in the control arm where the mortality rate was 4% and the daily rate (hazard) of being liberated from life-sustaining therapy was the same as observed within our German data. The intervention arm was then generated by multiplying the control arm daily rate of liberation by a fixed factor (hazard ratio). The mean days on life-sustaining therapy was then subtracted from 30 to obtain the free days. All estimates are based on simulating 10,000 samples of the required sample size, so power estimates will have more than a 95% chance of being accurate to within 1%. It derived from a virtual calculation, which showed that the distribution of the simulated control arm was nearly identical to the distribution of the actual observed German data. The calculation also provided the distribution of the intervention arm when the intervention causes a 20% relative increase in the daily rate of liberation from life-sustaining therapy compared to the control arm.

We expect that the sample size of the definitive trial will be approximately 700 per arm. This would provide about 90% power to detect a 20% relative increase in the daily rate of liberation from life-sustaining therapy. Such an effect size would result in a mean decrease of 1.5 days in the days of life-sustaining therapy from 6.8 to 5.3 days, or equivalently, a 1.5 day increase from 23.2 to 24.7 free days. We believe such an effect size is plausible and is in line with minimally clinically important differences accepted in other recent major trials in the ICU setting.

### Pilot study

Before advancing to a large-scale trial, a randomized, double-blind, placebo controlled pilot study of 80 patients will be carried out to determine the feasibility of a multicenter RCT, uncover problems regarding recruitment of patients, adherence with the study protocol and any contaminations (such as the co-application of selenium) and to develop study procedures for a large-scale RCT. The primary focus of the pilot study thus is to evaluate: 1) Recruitment of trial patients. Successful recruitment will be defined as >2 patients per month per site on average. A recruitment of 2 to 3 patients per month is considered as reasonable given the high numbers of competing studies in the field of cardiac surgery, missed patients and consent failure rates, all of which might limit our ability to enroll all of these potentially eligible patients. We expect to monitor success with recruitment throughout the study and make revisions to the recruitment procedures and eligibility criteria as necessary. 2.) Adherence to protocol. Successful adherence will be defined as ≥90% of prescribed intervention being administered across all patients. Preliminary estimates of non-administration of the trial intervention are needed, along with experience with behavioral strategies that maximize exposure to the intervention. 3.) Contamination. Success will be defined if <5% of patients have any non-study open-label intravenous selenium, in either group, during their hospital stay. Intravenous selenium is not currently the standard of care in any participating center but this outcome is important because contamination introduces risk of bias. A careful evaluation of the threat of contamination is key and mitigating strategies need to be identified.

After completion of this pilot trial, we will initiate the definitive randomized, double-blind, placebo controlled multicenter trial to evaluate our overall study hypothesis. Sites participating in the Canadian component of the multinational, multicenter pilot study include: University of Ottawa Heart Institute, Montreal Heart Institute and Sunnybrook Health Sciences Center. In addition, three European centers (RWTH Aachen University, Germany, University Hospital Frankfurt, Germany and Kiel University, Germany) will also be implementing the pilot study at their respective institutions. An additional objective of the pilot study is to finalize the primary outcome for the large-scale trial and develop estimates that can be used for a more precise calculation of the sample size for a large-scale trial. While a data safety monitoring committee has not yet been established for the pilot trial, it will be implemented for the definitive trial.

After completion of this pilot phase, descriptive and inferential statistics (mostly rates with 95% confidence intervals) will be presented to describe the pilot study feasibility outcomes. Safety and pharmacokinetic variables will be described by arm. However, due to the limited sample size and the potential of rolling the pilot study into the definitive study, clinical efficacy outcomes will be reported overall but not by arm. There are no planned interim or subgroup analyses for this pilot trial.

## Discussion

A significant number of patients require cardiac surgery for the management of their underlying heart disease. In addition to death, major morbidity from organ failure remains frequent following cardiac surgery, particularly in patients at high risk for complications and poor clinical outcomes. The perioperative inflammatory response as it occurs during the process in cardiac surgery is considered a major contributor to surgery-associated complications [3].

Various studies have previously demonstrated the crucial role of the essential trace element selenium within the multiples steps of antioxidant defense [8], and circulating selenium levels have been shown to correlate with the activity of GPx and other selenoenzymes in different clinical settings. In addition, selenium affects both the cell-mediated and the humoral immune defense mechanisms, and depressed selenium levels are associated with the reduction of natural killer cells [28, 29]. Interestingly the administration of sodium selenite is postulated to have a biphasic action, initially as a pro-oxidant and then as an antioxidant [30]. The early transient pro-oxidant effect of selenite might be a useful therapeutic strategy. In fact, an intravenous loading dose given as a bolus could have the following effects: 1) direct reversible inhibition of NF-κB binding to DNA controlling gene expression and thus downregulation of the synthesis of proinflammatory cytokines [31], 2) induction of apoptosis and cytotoxicity at the microcirculation level [32], and 3) a direct virucidal and bactericidal effect [30]. Wang *et al*. [33] demonstrated that a selenite loading bolus causes a transient peak plasma concentration of selenium and beneficial improvements in hemodynamics status, proinflammatory cytokines profile and survival. However, these findings have never been proven in ICU patients where plasma selenium significantly decreases [34].

In view of an impressive body of evidence that indicates a beneficial role of sodium selenite during inflammation, various preliminary clinical trials attempted to enhance the antioxidant and immunological defense mechanisms in critically ill patients by a sodium selenite supplementation strategy [15, 16, 35–38]. Accordingly, results from a recent meta-analysis indicated a significantly reduced incidence of infections and mortality in critically ill patients who had received a high dose (>500 μg) selenium supplementation during their ICU stay [28]. In the same way, Alhazzani *et al*. [39] and Huang *et al*. [40] very recently demonstrated reduced mortality in critically ill patients after selenium supplementation. While there is good evidence of benefit from selenium supplementation in critical illness, the optimal timing and dosage of selenium supplementation remains unclear.

A cardiac surgical setting with myocardial ischemia and reperfusion seems to be an ideal model to investigate the effect of perioperative sodium selenite supplementation. Indeed, two recently published studies investigated the effect of a perioperative sodium selenite supplementation in cardiac surgical patients. In a randomized trial, 117 patients received preoperative metabolic therapy, which consisted of several antioxidants (coenzyme Q10, magnesium, lipoic acid, omega-3 fatty acids and sodium selenite) and was compared to a placebo. In this study, the application of a metabolic cocktail resulted in reduced oxidative stress, less myocardial injury and reduced length of hospital stay [41]. In addition, results from a recently published open label trial, investigating the safety and pharmacokinetics of high-dose sodium selenite supplementation in cardiac surgical patients, demonstrated that the chosen doses were effective in preventing the intraoperative decrease of circulating selenium levels. When comparing sodium selenite-treated patients with a historical control group of cardiac surgical patients, the treatment group had less organ dysfunction, as assessed by the Sequential Organ Failure Assessment (SOFA) score on the first postoperative day. However, the loading dose was apparently not high enough to prevent the fall in circulating selenium levels to baseline levels on the first postoperative day. For this reason, the supplementation strategy in the present trial has been modified and a second loading dose of sodium selenite was added on admission to the ICU to compensate for this.

Pharmacokinetic studies suggest that optimal incorporation of supplemental selenium into selenoproteins requires approximately 72 hours [29], and there is some evidence to suggest that post-injury normalization of selenium levels is unable to significantly affect the incurred oxidative damage [42]. In this regard, we did consider including a preoperative oral sodium selenite supplementation in addition to our intravenous perioperative supplementation as part of a 2 × 2 factorial design. However, most participating cardiac centers report a high volatility in surgical booking and subsequent cancellation and re-scheduling (for example at University of Ottawa Heart Institute over a recent six month period 80% of the original elective and/or in-hospital urgent bookings were rescheduled from their original booked date (BM, personal communication)). Thus, it appeared highly likely that there would be major issues surrounding compliance and variability of any preoperative supplementation intervention, which would impact greatly on such a trial. In the current proposed pilot trial we will track timing and occurrences of screening, booking, cancellation and surgical dates to determine if and how a preoperative supplementation strategy might be feasible in a definitive trial.

In the present trial only high-risk cardiac surgical patients will be included. Previous data indicate that these patients are exposed to the most pronounced decrease of selenium during surgery [10], leading to the development of various organ dysfunctions. In addition, previous studies indicated that postoperative selenium blood levels were inversely correlated with duration of CPB, that is, the longer the surgical procedure, the more pronounced the postoperative decrease in circulating selenium levels [9, 10]. In addition, it was demonstrated that the preoperatively assessed EuroSCORE I inversely correlated to the postoperatively measured selenium levels (r = -0.312, *P* <0.01), indicating that this is the group of people who are most likely to benefit from perioperative selenium supplementation [9].

In conclusion, the SUSTAIN-CSX™-study is a prospective, randomized, double-blind, multicenter controlled multinational trial to investigate the effect of perioperative high-dose sodium selenite supplementation on the persistence of organ dysfunction in approximately 1,400 high-risk cardiac surgical patients.

## Trial status

Recruitment and enrollment of the first patients (pilot study) in both countries will start end of August 2014.

## Electronic supplementary material

Additional file 1: **Management of anesthesia and cardiopulmonary bypass: recommendations for general anesthesia and management of cardiopulmonary bypass in enrolled patients.** Intensive care unit and nutritional support: recommendations and rules for postoperative management of enrolled patients during the ICU stay with respect to extubation, discharge from ICU and nutrition. (DOCX 83 KB)

## Abbreviations

ADMA: Asymmetric Dimethyl-Arginin
AOX: Antioxidant
CPB: Cardiopulmonary bypass
EPA: Endogenous peroxidase activity
GMP: Good manufacture practice
GPX: Glutathione peroxidase
ICU: Intensive care unit
IL: Interleukin
LVAD: Left ventricular assist device
NF-kB: Nuclear factor-kappa b
POD: Persistent organ dysfunction
ROS: Reactive oxygen species
Sel-P: Selenoprotein P
SNP: Single nucleotide polymorphisms
TNFα: Tumor necrosis factor alpha.
