# Supplementary material for: SodiUm SeleniTe Adminstration IN Cardiac Surgery (SUSTAIN CSX-trial): study design of an international multicenter randomized double-blinded controlled trial of high dose sodium-selenite administration in high-risk cardiac surgical patients
Source: Trials. 2014 Aug 28;15:339. doi: 10.1186/1745-6215-15-339 (PMC4247649; doi:10.1186/1745-6215-15-339)
Supplement: Supplementary file 1 — Additional file 1: Management of anesthesia and cardiopulmonary bypass: recommendations for general anesthesia and management of cardiopulmonary bypass in enrolled patients. Intensive care unit and nutritional support: recommendations and rules for postoperative management of enrolled patients during the ICU stay with respect to extubation, discharge from ICU and nutrition. (DOCX 83 KB) [file 13063_2014_2316_MOESM1_ESM.docx]

**Additional files**

**Management of anesthesia and cardiopulmonary bypass**

All enrolled patients will receive standard perioperative care. Preoperative application of medication will follow the institutional practice (recommendation to stop metformin, ACE-inhibitors and AT_2_-receptor-antagonists) and are unlikely to influence the perioperative selenium levels.

Induction Anaesthesia is left to the discretion of the performing anaesthesiologist, which will follow the institutional routine. Furthermore the management of cardiopulmonary bypass as well will be performed in accordance to the institutional routine, which complies to the international recommendations (42).

**Intensive care unit and Nutritional support**

After completion of surgery, all patients will be transferred to the intensive care unit (ICU). Tracheal extubation will be performed when standard extubation criteria from the local institutional protocol are fulfilled. Patients will be discharged from the ICU after completion of the local standardized discharge criteria. In patients unable to be fed orally or extubated within the first 48 hours, enteral nutrition will be started within 24-48 at 10-25 ml per hour. Gastric residual volumes will be checked regularly and if less than 250-500 ml, the volume of infusion will be increased by 25 ml/hr until at goal rate (43). The target calorie intake (calculated for ideal body weight) is 25kcal·kg^-1^ per day and will be achieved within 2-3 days in all patients. Additional micronutrient supplements containing selenium or a simultaneous application of Vitamin C and the study medications will be prohibited and documented as a protocol violation.
